# A case of advanced hypopharyngeal cervical esophageal cancer treated by curative resection with management of tracheoesophageal fistula

**DOI:** 10.1007/s12328-023-01792-z

**Published:** 2023-04-08

**Authors:** Shinya Ohno, Yoshihiro Tanaka, Yuta Sato, Masahide Endo, Ryuichi Asai, Masahiro Fukada, Itaru Yasufuku, Naoki Okumura, Takao Takahashi, Nobuhisa Matsuhashi

**Affiliations:** Department of Gastroenterological Surgery, Pediatric Surgery, Gifu Graduate School of Medicine, Gifu University, 1-1 Yanagido, Gifu, 501-1194 Japan

**Keywords:** Esophageal cancer, Tracheoesophageal fistula, Prior tracheotomy

## Abstract

Advanced esophageal cancer with tracheal invasion is fatal due to airway narrowing and the possibility of tracheoesophageal fistula (TEF) formation during the treatment process. If a TEF develops, palliative care is often chosen. It is very rare that curative treatment is performed including with chemoradiotherapy (CRT) or surgery in such cases. A 71-year-old man presented with dysphagia. He was diagnosed as having hypopharyngeal and cervical esophageal cancer with severe airway stenosis (cT4b [main bronchus, thyroid] N3 M0 cStage IIIC), and we initially created a tracheostomy. Second, we chose induction chemotherapy to avoid fistula formation by CRT, but after one course of chemotherapy, he developed a TEF due to remarkable tumor shrinkage. We strictly managed both his airway and nutrition by continuous suctioning over the cuff of the tracheal cannula and prohibiting swallowing of saliva and enteral nutrition via nasogastric tube. After three courses of chemotherapy were administered, we performed pharyngo-laryngo-esophagectomy followed by adjuvant chemotherapy. The patient remains alive and recurrence free at 9 years postoperatively. In cases of upper TEF caused by advanced hypopharyngeal and cervical esophageal cancer, radical treatment may be possible by effective induction chemotherapy combined with strict airway and nutritional management after prior tracheostomy.

## Introduction

Advanced esophageal cancer with tracheal invasion is fatal not only due to airway narrowing but also the possibility of tracheoesophageal fistula (TEF) formation during the treatment process. Because of the poor prognosis of TEF, palliative care is often chosen [1]. We report the case of a patient with advanced hypopharyngeal and cervical esophageal cancer who developed TEF during induction chemotherapy but was successfully treated with complete resection under appropriate airway and nutritional management.

## Case report

**Fig. 1:**
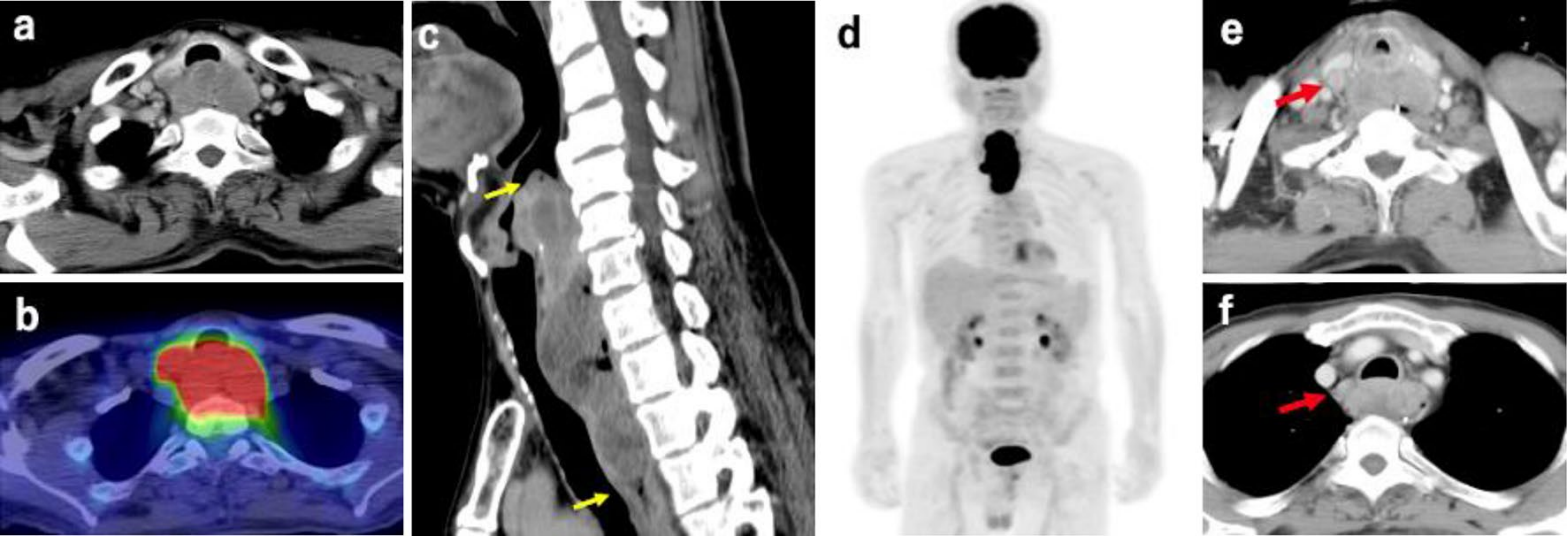
Contrast-enhanced CT and PET-CT findings. The tumor with entire circumferential wall thickening (**a**) with high 18F-fluorodeoxyglucose accumulation (**b**), which extended from the epiglottis to the upper thoracic esophagus (delineated by the yellow arrows) (**c**), invaded the trachea in the hypopharyngeal cervical esophagus, and no evidence of distant lymph node metastasis and other distant metastasis was found (**d**). There were, however, several metastases to regional lymph nodes (red arrows) (**e**, **f**) (colour figure online)

**Fig. 2:**
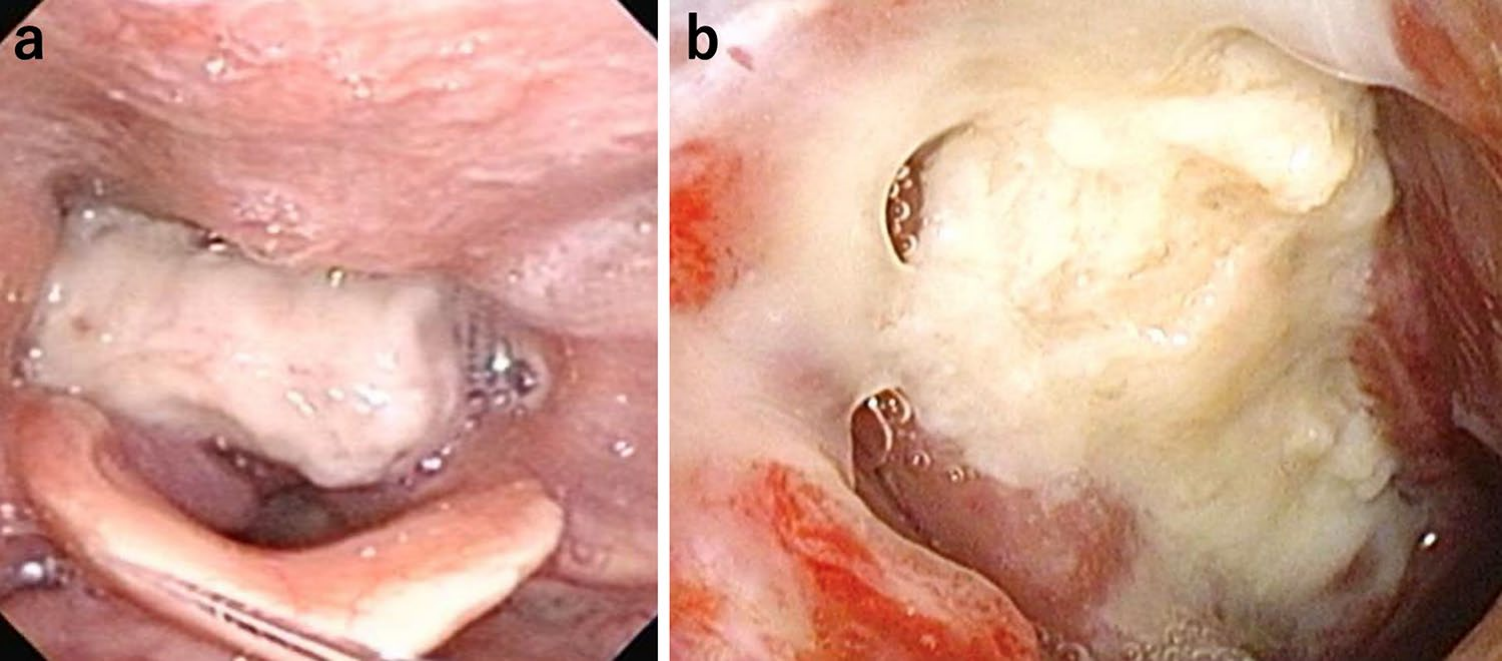
The laryngeal endoscopy (**a**) and bronchoscopy (**b**) findings. The tumor was exposed in the hypopharynx and invaded into the membranous portion, exposing the tumor in the trachea. The endoscope could not passed through

A 71-year-old man consulted his previous doctor with a chief complaint of dysphagia for three months. After his symptoms had developed, it became difficult to take a meal. He visited an otolaryngologist, and a tumor was located in the hypopharynx by laryngoscopy. A biopsy revealed squamous cell carcinoma. He developed dyspnea during a detailed examination and was rushed to his previous hospital. Esophageal stricture and airway stricture due to the tumor were observed, and he was immediately intubated and placed on a ventilator on the same day. Four days later, he underwent a tracheotomy and was transferred to our hospital for further treatment. On admission to our hospital, he was breathing spontaneously and his vital signs were stable. Laboratory findings showed a slightly elevated white blood cell count (12,800/mm^3^) and C-reactive protein level (2.61 mg/dL). His carcinoembryonic antigen was 1.3 ng/mL, carbohydrate antigen 19–9 was 13.3 U/mL, cytokeratin fragment was 0.9 ng/mL, and α-fetoprotein was 2.0 ng/mL, indicating no increase in the levels of these tumor markers. Contrast-enhanced computed tomography (CT) and positron emission tomography-CT revealed a tumor from the epiglottis to the upper thoracic esophagus with entire circumferential wall thickening, which invaded the trachea in the hypopharyngeal cervical esophagus, and no evidence of distant lymph node metastasis or other distant organ metastasis (Fig. 1a–d). However, several enlarged regional lymph nodes were present (Fig. 1e, f). Upper esophageal endoscopy showed that the tumor was exposed in the hypopharynx, and the fiberscope could not passed through (Fig. 2a). Bronchoscopy showed invasion into the membranous portion of the trachea, thus exposing the tumor in the trachea (Fig. 2b). The initial diagnosis of the tumor was T4b [main bronchus, thyroid] N3 M0 cStage IIIC according to the International Union Against Cancer Tumor–Node–Metastasis Classification of Malignant Tumors (7th edition) [2].

**Fig. 3:**
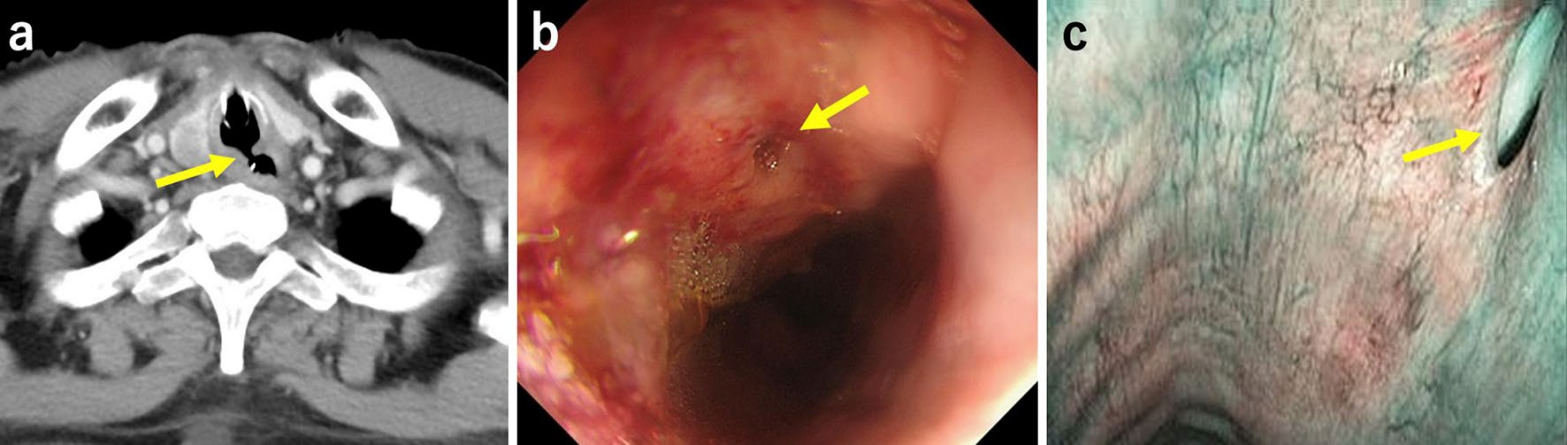
Contrast-enhanced CT (**a**), upper endoscopy (**b**), and narrow band imaging bronchoscopy (**c**) images after one course of DGS therapy. The primary tumor had shrunk, which caused formation of the cervical TEF (yellow arrows) (colour figure online)

**Fig. 4:**
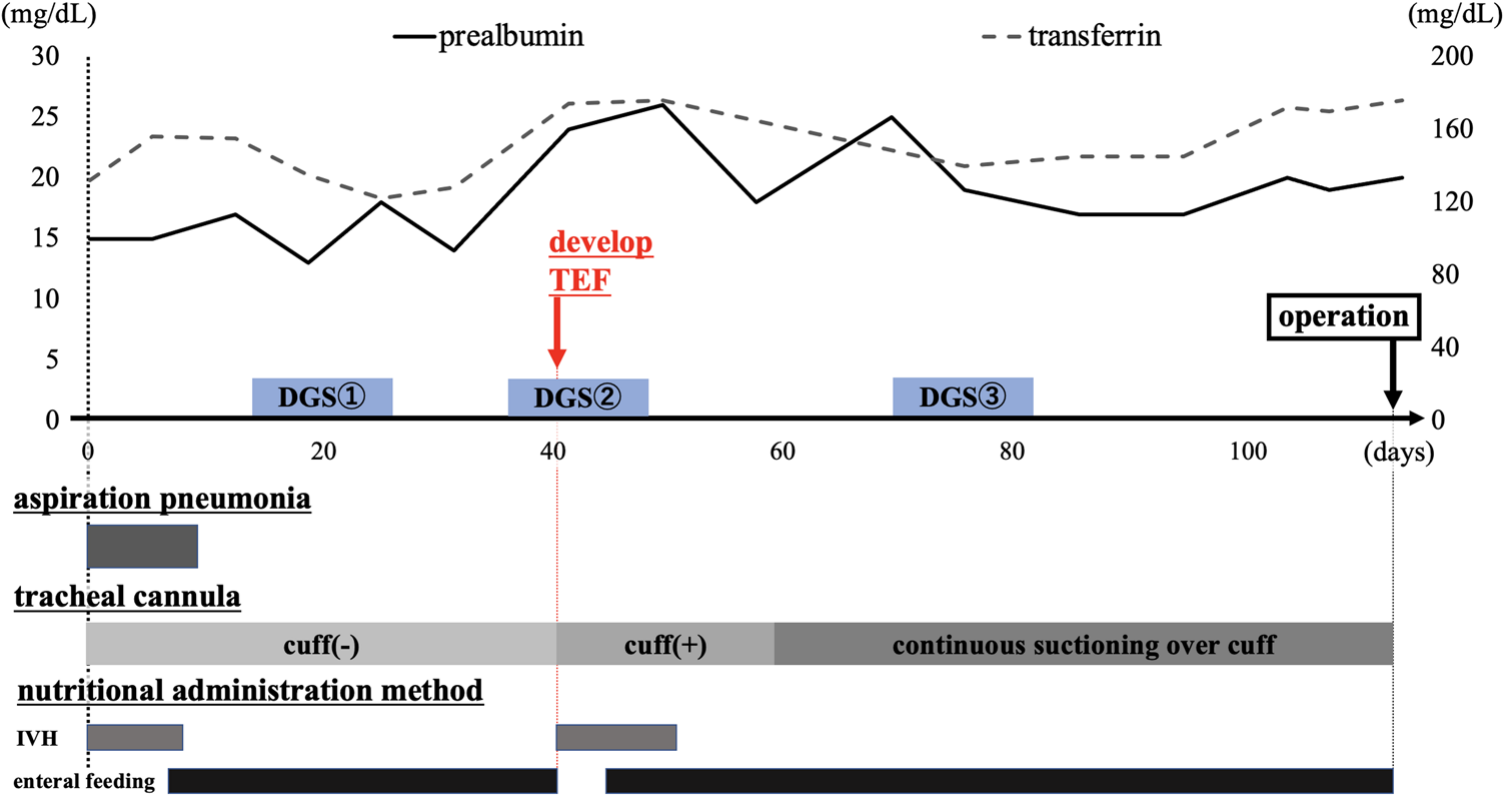
Preoperative hospitalization process. Nutritional status was assessed by rapid turnover protein. Nutritional management consisted of a combination of IVH and enteral nutritional supplementation at the onset of TEF and transiting entirely to enteral nutritional supplementation. Cuffing the tracheal cannula at the onset of TEF, and switched to continuous suctioning over the cuff due to pneumonia

**Fig. 5:**
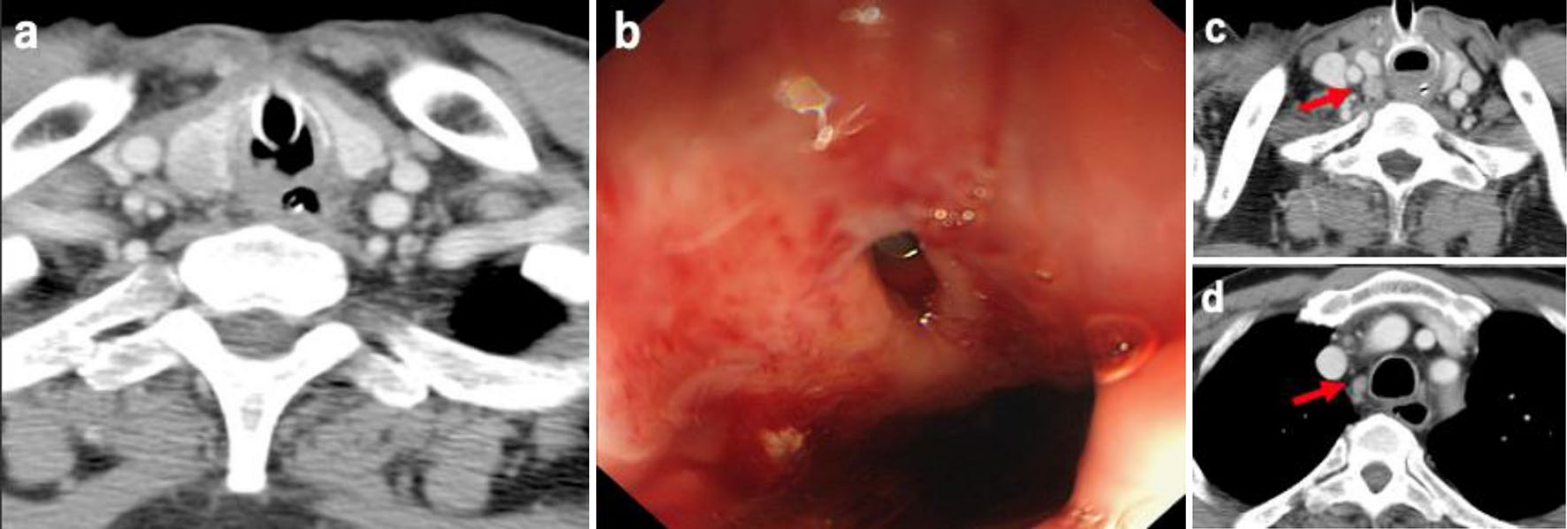
Contrast-enhanced CT and upper endoscopy images after three courses of DGS therapy. The primary tumor achieved complete response on the CT (**a**) and the TEF remained on the endoscopy (**b**). All enlarged regional lymph nodes were reduced by more than 30% in their long diameter (red arrows) (**c**, **d**) (colour figure online)

**Fig. 6:**
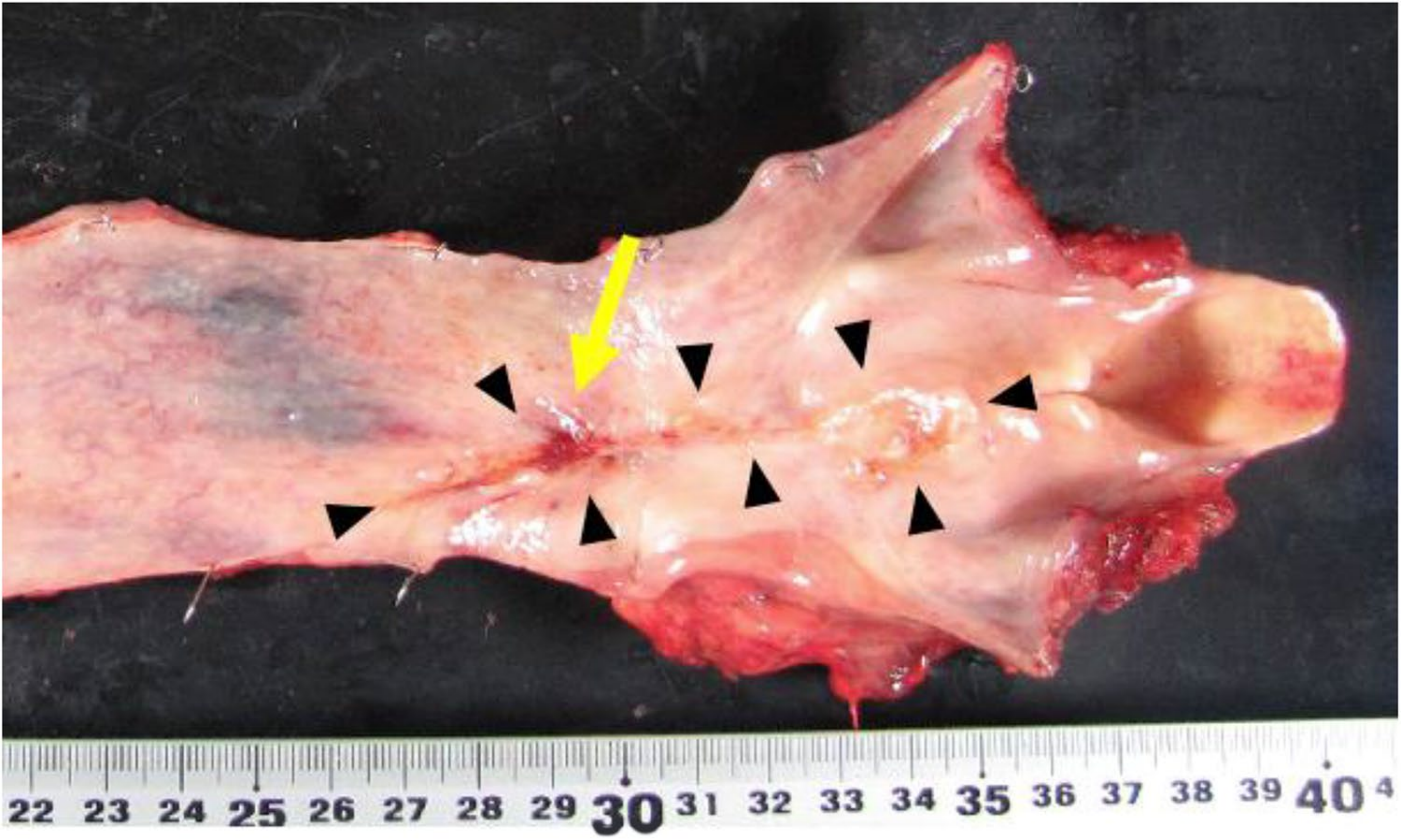
The resected specimen showed the remaining TEF (yellow arrow) in scar tissue (black arrowheads), but no gross tumor remained. Histopathological results showed no residual viable tumor cells in the primary tumor or lymph nodes (colour figure online)

Because of the findings of aspiration pneumonia in the lower lobe of the left lung on CT, the patient was started on chemotherapy instead of chemoradiotherapy (CRT), considering damage to the lung that would be caused by radiation. We initiated docetaxel, nedaplatin, and S-1 (DGS) therapy as induction chemotherapy [3, 4], intravenous hyperalimentation (IVH) via a right internal jugular vein catheter, and enteral nutritional supplementation via a nasogastric (NG) tube. After one course of DGS therapy, although the esophageal stricture was improved due to tumor shrinkage, a cervical TEF was suspected on contrast-enhanced CT (Fig. 3a). We confirmed the fistula by laryngeal endoscopy and bronchoscopy and diagnosed TEF (Fig. 3b, c). Because the tumor had shrunk markedly with chemotherapy and there was a possibility of TEF enlargement, we decided to continue chemotherapy with the goal of conversion surgery. Nutritional management was consisted of a combination of IVH and enteral nutritional supplementation at the onset of TEF, with a gradual increase of enteral nutritional supplementation after confirming the absence of nausea and vomiting, and finally transiting entirely to enteral nutritional supplementation. Nutritional status was assessed by rapid turnover protein. Airway management was consisted of cuffing the tracheal cannula, continuous suctioning over the cuff, and maintaining constant upper body positioning of at least 30 degrees to prevent aspiration. We managed the patient’s airway and nutrition strictly and administered three courses of DGS therapy (Fig. 4). Contrast-enhanced CT, upper endoscopy, and bronchoscopy showed that the primary tumor had achieved a clinically complete response and lymph node metastases showed a partial response (Fig. 5). Chemotherapy-related side effects of grade 3 neutropenia for which granulocyte-colony stimulating factor was used. A bronchoscopic biopsy was performed through the tracheostomy ostium preoperatively, and the biopsy of the bronchial membranous portion at the height of the 1st thoracic spine (5th tracheal cartilage) was negative for malignancy. We then performed conversion surgery comprising pharyngo-laryngo-esophagectomy, three-field lymphadenectomy, subtotal stomach reconstruction via the posterior mediastinal route, jejunostomy, total thyroidectomy, and permanent tracheostomy using a deltopectoral flap. The trachea was transected at the height of the 2nd thoracic spine (6th tracheal cartilage). The resected specimen showed the remaining TEF in scar tissue, but no gross tumor remained (Fig. 6). The patient’s postoperative course was uneventful. He started enteral feeding on postoperative day (POD) 2, was weaned from the ventilator on POD 4, started oral intake on POD 12, and was discharged on POD 30. Histopathological results showed no residual viable tumor cells in the primary tumor or lymph nodes, and he was evaluated as having a pathological complete response (pretreatment effect grade 3), and we achieved complete resection of the tumor. He was subsequently given two courses of DGS therapy as adjuvant chemotherapy, and he remains alive and recurrence free at 9 years postoperatively.

## Discussion

The esophagus is in close proximity to surrounding vital organs and vessels such as the trachea, aorta, and pulmonary veins. Advanced esophageal cancer frequently involves the trachea, with 20–36.6% of patients developing tracheal invasion [5, 6] and 5–10% developing TEF [7], a potentially fatal complication. TEF is generally more likely to occur after CRT for advanced esophageal cancer [8], and once it occurs, the prognosis is extremely poor, with an average survival time of about 1 month [9]. The most difficult aspect of TEF is the management of digestive fluids that flow into the trachea through the fistula, with most patients experiencing a fatal course due to repeated aspiration pneumonia during the course of their management.

A few cases of TEF due to benign or postoperative complications have been cured by ingenious surgical procedures such as using a muscle flap [8] or three-step surgery [10], but TEF caused by esophageal cancer is more complicated to treat because of the addition of cancer treatment. The treatment of TEF in esophageal cancer comprises a combination of chemotherapy or CRT for the tumor and stenting, esophageal bypass, or use of a muscle flap for TEF, all of which are often performed as palliative rather than curative treatment.

Esophageal stents and airway-esophageal double stents are often selected for TEF, especially to close the fistula and improve quality of life [11, 12]. Although there are reports of improved survival [13, 14], stenting remains a palliative treatment, and its disadvantages are the possibility of fistula enlargement and the difficulty of surgical manipulation due to stenting. Surgery may be an option, but most patients undergo esophageal bypass surgery [15, 16] or muscle flap coverage [9, 17] as palliative treatments. There are reports of two-step surgery for TEF caused by lung cancer [18] and TEF caused by malignant lymphoma that was closed after continued chemotherapy [19], but we were unable to find any reports of radical surgery for TEF caused by esophageal cancer.

Esmati E reported that patients who received induction chemotherapy experienced a trend toward superior survival for not only surgery but also CRT for cervical and upper thoracic esophageal cancers [20]. In Japanese guidelines for diagnosis and treatment of carcinoma of the esophagus 2022 [21], patients with T4 esophageal cancer are recommend to receive definitive CRT in a weak recommendation, besides induction chemotherapy is listed as an important treatment option for patients who have generally poor condition. Thus, no high-quality evidence exists for definitive CRT in patients with T4 esophageal cancer with comorbidities like aspiration pneumonia. Two reports showed that triplet chemotherapy regimen followed by surgery might controlled both local and systemic lesion [22, 23]. Thus, CRT might be recommended in patients with good general condition. We have chosen induction triplet regimen chemotherapy in our case because of easily miss swallowing. Our patient had aspiration pneumonia at the time of presentation, so we chose chemotherapy at first. Even when TEF occurred, we avoided radiation therapy because of fear of additional aspiration from TEF and damage to the lungs of radiation therapy.

For that reason, we kept on chemotherapy aiming surgery instead of radiation therapy against possibility of TEF enlargement.

At the time of a phase I dose-escalation study of DGS therapy, advanced cervical esophageal cancer was targeted [3], we selected DGS as the induction chemotherapy regimen. Induction chemotherapy enabled the complete resection including TEF as conversion surgery, which has resulted in 9-year recurrence-free survival at the present time.

**Fig. 7:**
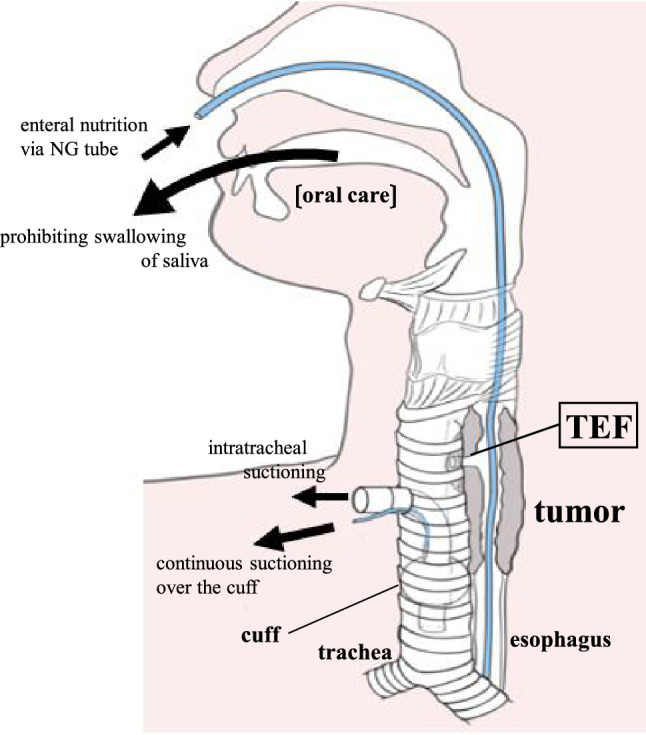
To prevent saliva and digestive fluid from entering the trachea, continuous suctioning over the cuff of the tracheal cannula and continuous suctioning of saliva in the oral cavity were performed. Enteral nutrition via a NG tube prevented backflow of digestive fluid into the fistula, *TEF* tracheoesophageal fistula, *NG* nasogastric

The prior tracheostomy allowed continuation of chemotherapy under strict airway and nutrition management. To safely administer chemotherapy to patients with TEF, it is necessary to reduce the risk of aspiration and pneumonia as much as possible by preventing backflow of digestive fluids into the fistula and collecting digestive fluids that have entered the trachea, such as by continuous suctioning over the cuff of the tracheal cannula, prohibiting swallowing of saliva, and maintaining constant upper body positioning. In addition, by encouraging regular expectoration of sputum through the tracheal cannula, nutritional management via enteral nutrition, oral care, and rehabilitation in collaboration with other medical staff [24], we were able to continue chemotherapy safely and without physical deterioration to the patient (Fig. 7).

Generally, creation of a permanent tracheostomy is considered if the trachea can be dissected cephalad of the suprasternal margin [25]. By performing biopsy of the cutting level of the trachea preoperatively by bronchoscopy through the tracheostomy site and determining the cutting level of the trachea to confirm a negative biopsy, we thereby ensured performance of complete resection of the tumor. Sakai et al. [26] also reported no difference in recurrence pattern in laryngeal cancer surgery even if a tracheotomy was performed first. If the TEF caused by the tumor is located caudal to the suprasternal margin, the tracheal cannula may not be long enough or a permanent tracheostomy may not be safely created, in which case stenting to close the fistula or esophageal bypass should be considered.

In the present case, a tracheostomy was performed first due to the presence of severe airway stenosis. Even in the absence of airway stenosis, however, prior tracheotomy may be advantageous with T4 cervical esophageal cancer. It facilitates confirmation of the fistula site, allows for better management of aspiration and pneumonia, and facilitates biopsy to determine the cutting level of the trachea when it comes to conversion surgery. A prior tracheostomy is also useful when a TEF develops.

In chemotherapy and CRT, many advantages of enteral nutrition have been reported, including maintenance of the intestinal mucosa (prevention of intestinal mucosal atrophy), maintenance of immunocompetence, maintenance of physiological functions of the digestive tract (intestinal peristalsis, digestive tract hormone secretion), avoidance of bacterial translocation, avoidance of bile stasis, and easy long-term management [27]. We provided enteral nutrition overnight during chemotherapy and used enteral and oral nutritional supplements after surgery [28, 29].

There are some limitations in this strict airway and nutrition management method. First, only patients with good comprehension can prohibit swallowing of saliva. Patients’ understanding and cooperation are essential for self-excretion of saliva. Prohibition swallowing of saliva cannot be implemented for patients with cognitive impairment. Second, to perform enteral nutrition, prolonged NG tube placement is necessary, which entails discomfort for the patient. We need to strive to alleviate the patient's discomfort using thin NG tubes such as 8Fr.

In advanced esophageal cancer, CRT is a good option for achieving a radical cure, but it is not sustainable when it causes a TEF. In upper TEF, surgical en-bloc resection of the trachea and esophagus in the region of the TEF is possible, and radical surgery may be possible with induction chemotherapy. In the present case, chemotherapy was chosen instead of CRT because of the patient’s initial pneumonia. However, even if a TEF develops during CRT, radical surgery may be possible after adequate chemotherapy with appropriate systemic management, and in such cases, it may be advisable to switch to a triple-drug regimen with the expectation of greater efficacy [3].

In conclusion, advanced hypopharyngeal and cervical esophageal cancer with upper TEF may be treated with chemotherapy and curative surgery while strictly managing the TEF by a prior tracheostomy.

## Abbreviation

TEF: Tracheoesophageal fistula
CT: Computed tomography
CRT: Chemoradiotherapy
DGS: Docetaxel, nedaplatin and S-1
IVH: Intravenous hyperalimentation
NG: Nasogastric
POD: Postoperative day
